# Clinical application of noninvasive chromosomal screening for elective single-blastocyst transfer in frozen-thawed cycles

**DOI:** 10.1186/s12967-022-03640-z

**Published:** 2022-12-03

**Authors:** Rui Chen, Ni Tang, Hongzi Du, Yaxin Yao, Yangyun Zou, Jing Wang, Dunmei Zhao, Xueliang Zhou, Yang Luo, Lei Li, Yuling Mao

**Affiliations:** 1 Department of Obstetrics and Gynecology, Center for Reproductive Medicine, Guangdong Provincial Key Laboratory of Major Obstetric Diseases, The Third Affiliated Hospital of Guangzhou Medical University, Guangzhou, China; 2 Key Laboratory for Reproductive Medicine of Guangdong Province, The Third Affiliated Hospital of Guangzhou Medical University, Guangzhou, China; 3 Department of Clinical Research, Yikon Genomics Company, Ltd, Suzhou, 215000 China

**Keywords:** Noninvasive chromosomal screening (NICS), Elective single embryo transfer (eSET), Frozen-thawed cycles

## Abstract

**Background:**

The objective of this study was to explore the clinical application of noninvasive chromosomal screening (NICS) for elective single-blastocyst transfer (eSBT) in frozen-thawed cycles.

**Methods:**

This study retrospectively analysed the data of 212 frozen-thawed single-blastocyst transfers performed in our centre from January 2019 to July 2019. The frozen embryos were selected based on morphological grades and placed in preincubation for 6 h after warming. Then spent microdroplet culture media of frozen-thawed blastocysts were harvested and subjected to NICS. The clinical outcomes were evaluated and further stratified analysis were performed, especially different fertilization approaches.

**Results:**

The clinical pregnancy, ongoing pregnancy, and live birth rates in the euploidy group were significantly higher than those in the aneuploidy group (56.2% *versus* 29.4%) but were nonsignificantly different from those in the chaotic abnormal/NA embryos group (56.2% *versus* 60.4%). Compared with day6 (D6) blastocysts, D5 blastocysts had a nonsignificantly different euploidy rate (40.4% versus 48.1%, *P* = 0.320) but significantly increased clinical pregnancy (57.7% versus 22.2%, *P* < 0.001), ongoing pregnancy (48.1% versus 14.8%, *P* < 0.001), and live birth rates (48.1% versus 13.0%, *P* < 0.001). The percentage of chaotic abnormal/NA embryos group was significantly higher among D5 embryos than among D6 embryos (30.1% versus 11.1%, *P* = 0.006). The percentage of aneuploid embryos was higher among the embryos with lower morphological quality(21.5% among ‘good’ embryos versus 34.6% among ‘fair’ embryos versus 46.0% among ‘poor’ embryos, *P* = 0.013); correspondingly, the overall clinical pregnancy, ongoing pregnancy and live birth rate rates showed similar declines.

**Conclusions:**

NICS combined with morphological assessment is an effective tool to guide frozen-thawed SBT. The optimal embryo for SBT is a ‘euploid embryo with good morphology’, followed sequentially by a ‘chaotic abnormal/NA embryo with good morphology’, ‘euploid embryo with fair morphology’, and ‘chaotic abnormal/NA embryo with fair morphology’.

**Supplementary Information:**

The online version contains supplementary material available at 10.1186/s12967-022-03640-z.

## Background

In vitro fertilization-embryo transfer (IVF-ET) refers to an assisted reproductive technology in which gametes (sperms and eggs) are collected from ovaries and fertilized under in vitro conditions to form embryos, and then high-quality embryos are implanted in a uterus to develop into foetuses. To date, more than eight million babies have been born worldwide as a result of IVF-ET. Multiembryo transfer leads to a high multiple pregnancy rate up to 20% [1]. Multiple pregnancies may increase the risk of adverse pregnancy outcomes and endanger maternal and infant health. Elective single embryo transfer (eSET) has been increasingly used worldwide as the most effective method to reduce the rate of multiple pregnancies [2]. However, the success rate of SET has been limited mainly by the lack of scientific methods evaluating the developmental potential of embryos.

Currently, the most commonly used method for embryo selection is morphological assessment. However, nearly 50% of embryos with good morphology are aneuploidy, suggesting that morphology alone is insufficient for chromosomal assessments of embryos [3, 4]. Embryonic aneuploidy is an important cause that decreases the pregnancy rate and increases the miscarriage rate in IVF. Studies have confirmed that aneuploidy can cause developmental arrest and implantation failure of embryos [5]. Embryonic aneuploidies are responsible for more than 50% of abortions [6]. Clinically, for patients with a high risk of producing aneuploidy embryos, such as women of advanced age and women who have experienced recurrent miscarriages or multiple implantation failures [7, 8], an approach that helps avoid aneuploid embryo transfer is using preimplantation genetic testing for aneuploidy (PGT-A) to analyse the chromosome copy number before implantation. However, for patients who did not undergo PGT-A in a fresh cycle and had implantation failure or miscarriage after fresh embryo transfer, the only option is to select frozen embryos based on morphological assessment, which cannot determine the status of chromosomes. For these patients, PGT-A of frozen embryos requires a series of procedures including thawing, biopsy, and refreezing. In particular, embryo biopsy is invasive. Embryo biopsy requires special equipment and well-trained professionals and may have a negative impact on the embryo’s ability to develop and implant [9, 10]. In addition, a long-term follow-up must be performed due to offspring safety concerns related to the biopsy cycle [11, 12]. Therefore, if the chromosome ploidy of embryos can be detected by non-invasive chromosome screening (NICS), the embryo biopsy will be avoided after thawing, which reduces the possibility of embryo damage.

Since Stigliani et al. [13–16] first discovered that embryos release cell‐free DNA (cfDNA) into culture medium during culture, noninvasive PGT-A using cfDNA has become a research hotspot in the field of assisted reproduction. In particular, studies on the culture media of frozen-thawed embryos showed that compared with fresh embryo culture medium, frozen embryo culture medium was a more suitable material for niPGT-A. In previous studies, after frozen embryos were thawed and cultured for 14–24 h, the culture medium or a mixture of blastocoel fluid and culture medium were collected and yielded a cfDNA amplification success rate of 92.3–100%. Taking the whole-embryo results as a gold standard for comparison, the results from culture medium were highly concordant and reached an accuracy of 87–100% [17–20]. Kuznyetsovet al. [17] reported that the concordance rate between noninvasive chromosomal screening results and whole-embryo results was even higher than that between trophectoderm (TE) results and whole-embryo results (96.4% versus 87.5%). However, the duration of embryo thawing and culture was relatively long (14–24 h) in the studies above compared to that in frozen-thawed cycles (generally two to three hours) [21, 22]. In addition, considering that a test for copy number variation (CNV) requires 9 h [23], the total in vitro culture time would be 23–33 h, which is too long for frozen blastocysts that have met the freezing requirements to be transferred within the optimal time window. Therefore, the approach applied in the studies above may reduce the embryo implantation success rate and is not suitable for application in clinical practice.

In this observational study, a clinically implementable embryo thawing and culture method for single-blastocyst transfer (SBT) was used, where frozen embryos selected according to morphological grades were thawed and cultured in 15–20 µL of culture medium for 6 h. The patients were followed up for clinical outcome evaluations. Meanwhile, the culture media of blastocysts were collected for NICS, and the relationship between NICS results and the clinical outcomes of patients was compared to explore whether NICS results can be used to effectively assess the developmental potential of frozen-thawed embryos. The present study included 212 IVF or ICSI patients who underwent frozen-thawed SBT at our centre and represents the first large-scale retrospective study analysing the relationship between NICS results and clinical outcomes in frozen-thawed SBT.

## Methods

### Study design and subjects

This retrospective cohort study enrolled 212 patients who underwent elective SBT (eSBT) in frozen-thawed cycles from January 2019 to July 2019 in the Reproductive Medicine Center of the Third Affiliated Hospital of Guangzhou Medical University. The frozen embryos to be transferred were selected based on their morphological grades according to Gardner and Schoolcraft's grading system [24], and the culture media of 212 blastocysts were collected for analysis. The inclusion criterion was that patients had frozen embryos and agreed to receive SBT using a frozen embryo selected according to morphological quality. The exclusion criteria were as follows: (1) patients with hydrosalpinx who did not undergo proximal tubal ligation; (2) patients with endocrine disease, infectious disease, or immune dysfunction; and (3) patients with intimal polyps who were not treated prior to embryo thawing and implantation. The study was approved by the Ethics Committee (Ethics No. Bioethical Review [2019] No. 003), and all patients signed informed consent forms.

All 212 patients underwent SBT using frozen embryos selected according Gardner and Schoolcraft grading system. The selected frozen embryos that had been thawed and cultured for 6 h were implanted in the patients, and their microdroplet culture media were frozen and stored for NICS. Patients were followed up until a live birth was achieved. The relationship between NICS results and clinical outcomes was analysed.

### SCM collection and testing

#### Blastocysts vitrification and warming

Vitrification and warming of blastocysts were performed using the method according to the kit manufacturers’ specifications (Kitazato BioPharma Co., Ltd., Japan). Before vitrification, the laser assisted hatching system was used to artificially shrink the cystic cavity away from the inner cell mass to release the blastocyst fluid, and vitrification was performed after it is completely shrunk. The freezing process was carried out at 37 °C for 2 min in ES solution and 45–60 s in VS solution (solutions were included in the kit), then the embryos were placed on the top of the Cryotop, and immediately put into liquid nitrogen. During blastocyst recovery, the Cryotop loaded with blastocysts was taken out from liquid nitrogen, quickly put into TS that had been equilibrated to 37 °C for 1 min. And then transferred to DS, WS1 and WS2 equilibrated at room temperature in turn, 3 min each step. The thawed blastocysts were cultured in 20 µl G2.5 PLUS media microdrop (Vitrolife) for 6 h under 6% CO_2_, 5% O_2_, and 89% N_2_. Blastocoel expansion was considered to be suitable for implantation.

#### Culture medium collection

Blastocyst was transferred from 20 µl microdrop culture medium (Vitrolife) to transplantation dish before embryo transfer. Then the spent microdrop culture medium was collected, and the blank culture solution was collected as control at the same time. The microdrop culture medium was transferred to DNase-free PCR tubes containing 5 µL cell lysis buffer (Xukang Medical Technology (suzhou) Co., Ltd, China) and stored at stored at− 80 °C prior to analysis.

#### Amplification、library construction and sequencing

A ChromInst^™^ (EK100100724, NICSInst™ Library Preparation Kit, Xukang Medical Technology (suzhou) Co., Ltd, China) was used to conduct whole-genome amplification (WGA). Quality control of the NGS libraries was performed using Qubit 3.0 and 1.5% agarose gel electrophoresis. Sequencing was conducted using the Illumina platform, HiSeq 2500 (Illumina, San Diego, CA, USA), yielding ∼2 million sequencing reads (single-end, 55 bp) on each sample [19, 20, 25–27]. We sequenced the amplified genome of each sample at the depth × 0.036. The details of WGA and sequencing were described previously [19].

#### CNV analysis

The data were analyzed and visualized using ChromGo^™^ Analysis Software (Xukang Medical Technology (suzhou) Co., Ltd) with default parameters. The operation of the software was described in the Huang’s article [27, 28]. High-quality reads were extracted and mapped to the human hg19 genome. After removing duplicate reads, the high-quality read numbers were counted along the whole genome with a bin size of 1 Mb and normalized by the GC content and a reference dataset. The circular binary segmentation (CBS) algorithm was used to detect CNV segments. The coefficient of variation (CV), calculated as the ratio of the standard deviation of read density to its average, was used to assess the amplification success. A CV value of less than 0.2 was considered as a successful amplification. If the result indicated mosaicism, the embryo was initially classified as ‘euploid’ when the extent of mosaicism was below 30% and as ‘aneuploid’ when the extent of mosaicism was above 30%, with a detection limit for segmental aneuploidy of ≥ 10 Mb. > 5 chromosome aneuploidies were considered as chaotic abnormal. Taking into account the reference of sequencing result analysis, etc. and no uniform standard of cutoff value, so we finally followed the reporting standard 30% of PGT-A in our center. All embryos are divided into euploid group, aneuploidy group and chaotic abnormal/not available (NA) group according to NICS results [29].

### Outcome measure

#### Outcomes and follow-up

The end point of follow-up was the live birth. Clinical pregnancy was defined as one gestational sacs and fetal heartbeat in the uterine cavity confirmed by ultrasound at 28–30 days after transplantation. Ongoing pregnancy was defined as a detectable fetal heart after 12 weeks of gestation. Early miscarriage was defined as the loss of clinical pregnancy (at least one gestational sac) by ultrasound before 12 weeks of gestation. Live birth was defined as the delivery of 1 living infants at greater than 28 weeks’ gestation.

#### Data calculation formula

Clinical pregnancy rate = number of clinical pregnancy cycles/number of frozen-thawed cycles, Ongoing pregnancy rate = number of ongoing pregnancy cycles/number of frozen-thawed cycles, Miscarriage rate = number of miscarriage cycles/number of clinical pregnancy cycles, live birth rate = number of live births/the number of frozen-thawed cycles.

### Statistical analysis

Data analyses were conducted using SPSS version 19 (IBM, Armonk, NY, USA). Continuous variables were analyzed using one-way analysis of variance if normally distributed (as verified by the Shapiro–Wilk test) or the Kruskal–Wallis H test if nonnormally distributed. Categorical data are expressed as counts and percentages and were determined to be statistically significant using the chi-square test or Fisher’s exact test. Multiple logistic regression analysis was conducted to compare the outcomes of euploid group, aneuploidy group and chaotic abnormal/NA group after controlling the covariables at p < 0.10 and covariables considered clinically influential. A two-sided P-value equal to or less than 0.05 was considered to be statistically significant.

## Results

### Study workflow and baseline data of the subjects

**Fig. 1:**
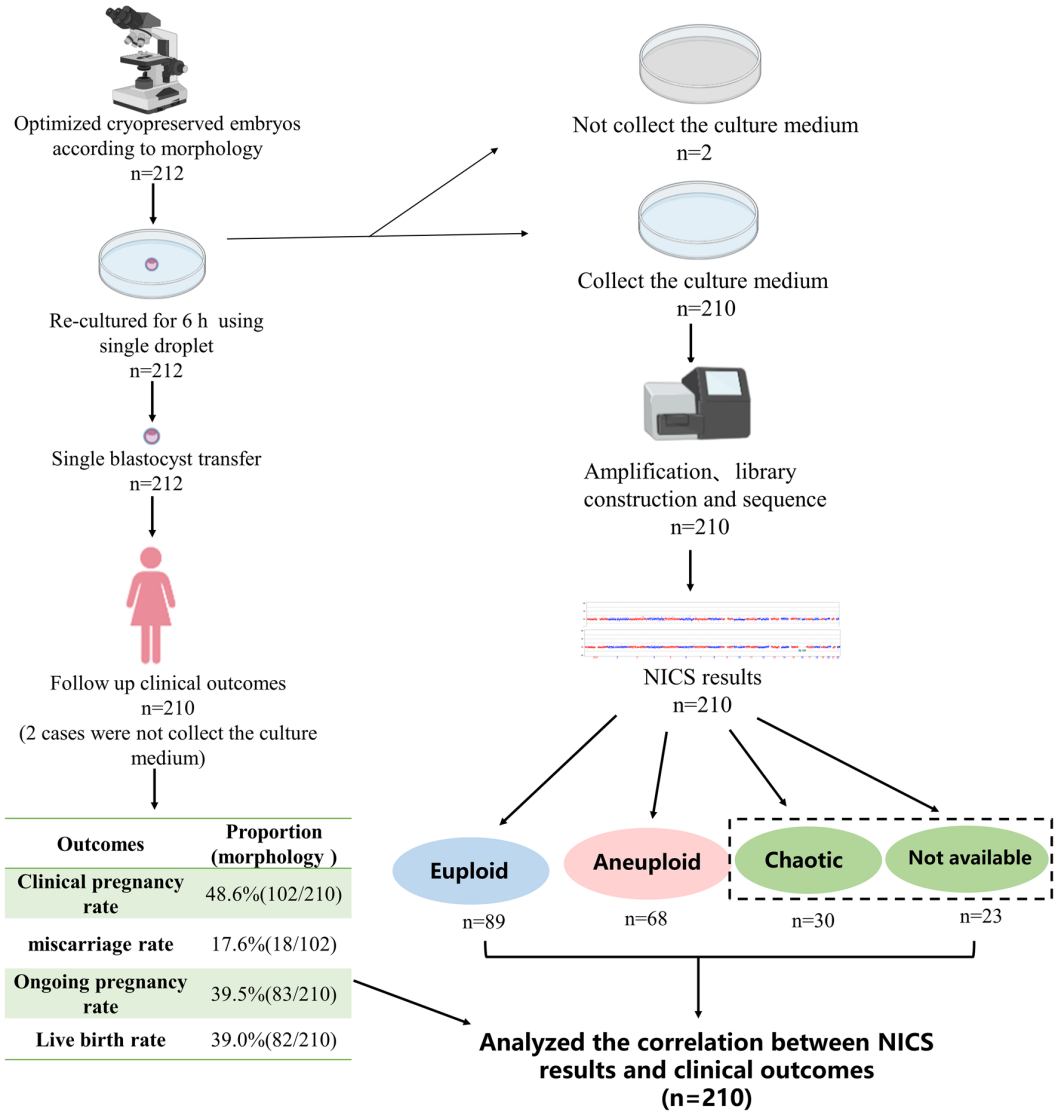
Flowchart of the study. Patients implanted with single frozen blastocysts selected based on morphological quality

This study included 212 frozen blastocysts selected according to morphological quality. Following routine procedures in our centre, a thawed blastocyst was cultured in single microdrops for 6 h and then transferred to a transplantation dish for SBT, which was performed under ultrasound guidance. The microdroplet culture medium was collected for NICS, and the patient was followed up for clinical outcome evaluation. Ultimately, only 210 patients were included in the final analysis because the spent culture media (SCM) of two embryos were not obtained. NICS results showed that 23 SCM samples failed in WGA, 30 failed the quality test by gene sequencing, 89 showed euploidies, and 68 showed aneuploidies (Fig. 1).

Table 1 shows the baseline clinical characteristics of all 210 patients, with the patients divided into the euploidy, aneuploidy, and chaotic abnormal/NA embryo groups according to NICS results. The baseline characteristics included both male and female ages, body mass index (BMI), years of infertility, fertilization approaches, infertility type, the cause of infertility, embryonic days, and the morphological grade of the blastocyst. According to the classification standards described in Munne’s article, the morphology of embryos was divided into three levels of good, fair, and poor, which included the grades of AA/BA/AB, BB/AC, and CA/BC/CB, respectively [30]. The three groups did not show statistically significant differences in most characteristics except for both male and female ages, fertilization approaches, whether infertility was primary or secondary, embryonic days, and the morphological grade of the blastocyst (Table 1).

**Table 1:** Baseline clinical characteristics of the patients

Characteristic	Euploidy group	Aneuploidy group	chaotic abnormal/NA embryo group	Overall	P-value
Number of patients, n	89	68	53	210	
Age of female, mean ± SD, y	30.9 ± 4.0	33.0 ± 4.7	30.6 ± 3.2	31.5 ± 4.2	0.001
BMI of female, mean ± SD, kg/m^2^	21.7 ± 3.5	22.6 ± 3.5	22.0 ± 3.8	22.1 ± 3.6	0.355
Age of male, mean ± SD, y	33.5 ± 5.0	35.1 ± 5.1	32.9 ± 3.9	33.9 ± 4.8	0.033
BMI of male, mean ± SD, kg/m^2^	24.3 ± 3.7	24.2 ± 3.2	25.0 ± 3.5	24.5 ± 3.5	0.430
Infertility duration, mean ± SD, y	3.9 ± 2.8	4.3 ± 3.4	4.1 ± 2.5	4.1 ± 2.9	0.690
Types of infertility					
Primary (%)	62.9% (56/89)	42.6% (29/68)	64.2% (34/53)	56.7% (119/210)	0.018
Secondary (%)	37.1% (33/89)	57.4% (39/68)	35.8% (19/53)	43.3% (91/210)	
Indication					
Male factor (%)	29.2% (26/89)	26.5% (18/68)	39.6% (21/53)	31.0% (65/210)	0.306
Female factor (%)	34.8% (31/89)	38.2% (26/68)	39.6% (21/53)	37.0% (78/210)	
Both (%)	36.0% (32/89)	35.3% (24/68)	20.8% (11/53)	31.9% (67/210)	
Approaches of fertilization					
ICSI (%)	57.3% (51/89)	36.8% (25/68)	43.4% (23/53)	47.1% (99/210)	0.031
IVF (%)	42.7% (38/89)	63.2% (43/68)	56.6% (30/53)	52.9% (111/210)	
Embryonic days					
D5 (%)	70.8% (63/89)	67.6% (46/68)	88.7% (47/53)	74.3% (156/210)	0.019
D6 (%)	29.2% (26/89)	32.4% (22/68)	11.3% (6/53)	25.7% (54/210)	
Quality grade					
Good (AA/BA/AB) (%)	37.1% (33/89)	25.0% (17/68)	54.7% (29/53)	37.6% (79/210)	0.005
Fair (BB/AC) (%)	38.2% (34/89)	41.2% (28/68)	35.8% (19/53)	38.6% (81/210)	
Poor (CA/BC/CB) (%)	24.7% (22/89)	33.8% (23/68)	9.4% (5/53)	23.8% (50/210)	

### NICS results and clinical outcomes

Among 210 patients, one ectopic pregnancy and one late miscarriage were noted. The clinical pregnancy rate was 48.6% (102/210), the ongoing pregnancy rate was 39.5% (83/210), the early miscarriage rate was 17.6% (18/102), and the live birth rate was 39.0% (82/210) (Fig. 1). The CNV results of abortion samples from six patients were shown in Additional file 1: Table S1. In two cases, the NICS results were classified as chaotic abnormal/NA (> 5 chromosome aneuploidies), and the abortions were euploid. One case of abortion is euploid, but NICS judged as aneuploid due to the 5 chromosome aneuploidies, which was just at the critical value.

**Fig. 2:**
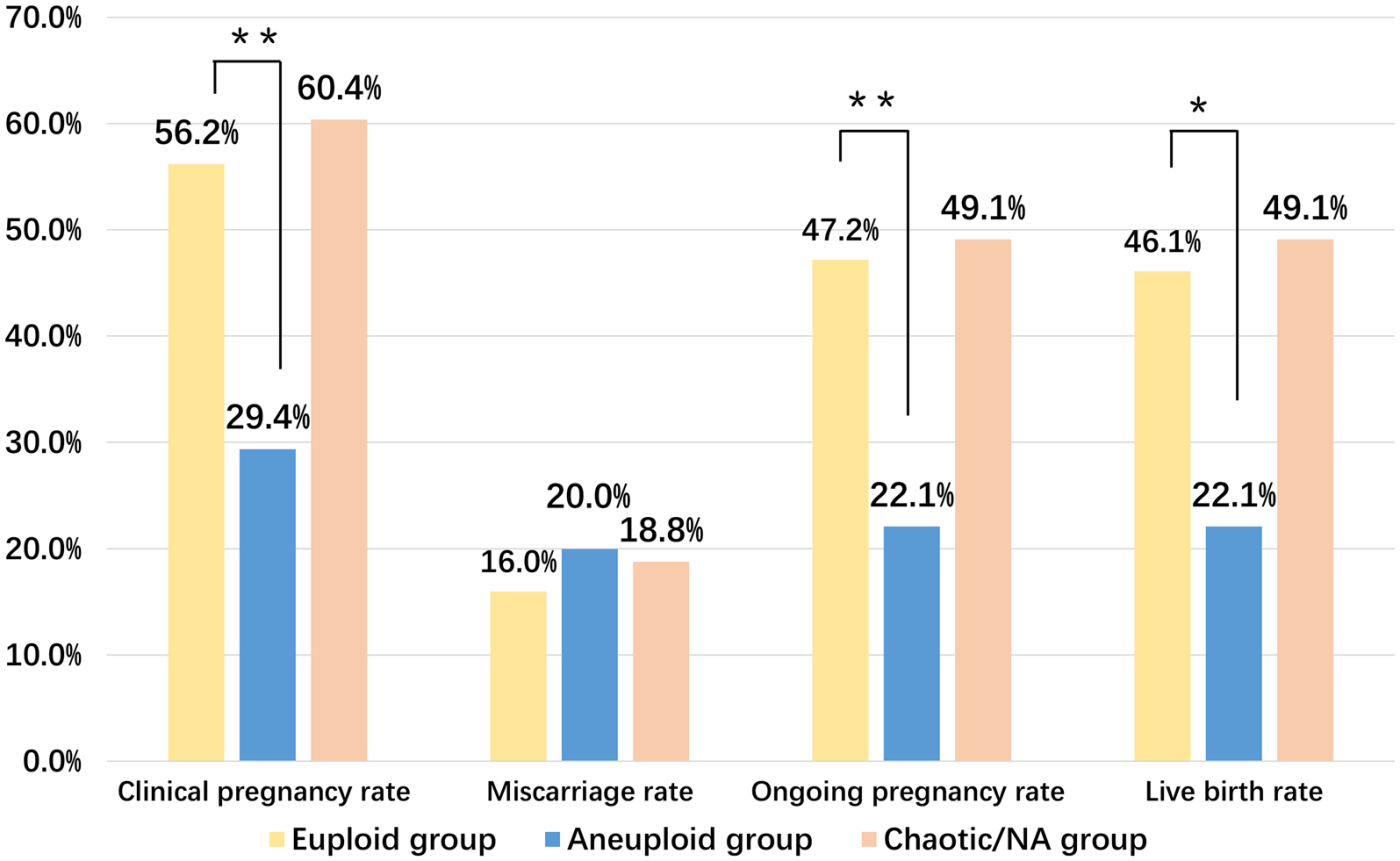
Comparison of clinical outcomes between the euploidy group, aneuploidy group, and chaotic abnormal/NA embryo group

Both male and female ages, morphological grade, embryonic days, fertilization approaches, and infertility type were independent variables in the binary logistic regression analysis. The results showed that compared with the aneuploidy group, the euploidy group had a statistically significantly higher clinical pregnancy rate (56.2% versus 29.4%, adjusted odds ratio (OR) 0.33, 95% confidence interval (CI) 0.15–0.72), ongoing pregnancy rate (47.2% versus 22.1%, adjusted OR 0.34, 95% CI 0.15–0.77), and live birth rate (46.1% versus 22.1%, adjusted OR 0.39, 95% CI 0.18–0.86) (Fig. 2 and Additional file 1: Table S2).

Compared with the chaotic abnormal/NA embryo group, the euploidy group did not show any significant difference in the clinical pregnancy rate (56.2% versus 60.4%, adjusted OR 0.85, 95% CI 0.39–1.85), ongoing pregnancy rate (47.2% versus 49.1%, adjusted OR 0.76, 95% CI 0.36–1.63), and live birth rate (46.1% versus 49.1%, adjusted OR 0.78, 95% CI 0.37–1.67) (Fig. 2 and Additional file 1: Table S2).

### Stratified analysis exploring the factors affecting clinical outcomes

Further analysis was stratified by female age, morphological grade, and embryonic days because these three characteristics showed significant differences (*P* < 0.05) and were also known factors affecting clinical outcomes.

According to female age, the patients were divided into two groups for stratified analysis: < 35 years old and ≥ 35 years old. The results showed that compared to the ≥ 35 age group, the < 35 age group had a higher embryonic euploidy rate (45.2% versus 31.1%) and lower embryonic aneuploidy rate (26.2% versus 57.1%). The < 35 age group had a significantly higher overall clinical pregnancy rate (55.4% versus 21.4%,* P* < 0.001), ongoing pregnancy rate (45.8% versus 14.3%,* P* < 0.001), and live birth rate (45.2% *versus* 14.3%,* P* < 0.001), but the miscarriage rate was not markedly different from that of the ≥ 35 age group (16.1% versus 33.3%,* P* = 0.194). Within the < 35 age group, the differences in clinical pregnancy, ongoing pregnancy rates and live birth retes among the three group were statistically significant (*P* = 0.034, *P* = 0.041 and *P* = *0.050*) (Table 2 and Additional file 1: Table S3). Within the ≥ 35 age group, the clinical pregnancy, ongoing pregnancy, and live birth rates were higher in the euploidy group than in the aneuploidy group, but the differences were statistically nonsignificant (Table 2).

**Table 2:** Female age-stratified comparison of clinical outcomes

Age of female	NICS results	Ratio	Clinical pregnancy rate	Miscarriage rate	Ongoing pregnancy rate	Live birth rate
< 35 years old (n = 168)	Euploid	45.2% (76/168)	60.5% (46/76)	15.2% (7/46)	51.3% (39/76)	50.0% (38/76)
	Aneuploid	26.2% (44/168)	38.6% (17/44)	17.6% (3/17)	29.5% (13/44)	29.5% (13/44)
	Chaotic/NA	28.6% (48/168)	62.5% (30/48)	16.7% (5/30)	52.1% (25/48)	52.1% (25/48)
	p-value		0.034	0.999	0.041	0.050
≥ 35 years old (n = 42)	Euploid	31.0% (13/42)	30.8% (4/13)	25.0% (1/4)	23.1% (3/13)	23.1% (3/13)
	Aneuploid	57.1% (24/42)	12.5% (3/24)	33.3% (1/3)	8.3% (2/24)	8.3% (2/24)
	Chaotic/NA	11.9% (5/42)	40.0% (2/5)	50.0% (1/2)	20.0% (1/5)	20.0% (1/5)
	p-value		0.241	0.999	0.344	0.344
P value for age subgroups		< 0.001	< 0.001 (55.4%vs21.4%)	0.194 (16.1%vs 33.3%)	< 0.001 (45.8%vs 14.3%)	< 0.001 (45.2%vs 14.3%)

According to the morphological grades of embryos (good, fair, or poor), the patients were divided into three groups for stratified analysis. The euploidy rates in the good, fair, and poor morphology groups were 41.8%, 42%, and 44.0%, respectively. The aneuploidy rates were 21.5%, 34.6%, and 46.0% in the good, fair, and poor morphology groups, respectively; in other words, worse embryo morphology corresponded to a higher aneuploidy rate. The overall clinical pregnancy, ongoing pregnancy, and live birth rates decreased with deterioration of embryo morphology (59.5% versus 44.4% versus 38.0%,* P* = 0.038; 49.4% versus 39.5% versus 24.0%,* P* = 0.016; 49.4% versus 39.5% versus 22.0%,* P* = 0.008, respectively). Embryos with ‘poor’ morphology led to a higher miscarriage rate (31.6%) compared with those of the good morphology group (17.0%) and the fair morphology group (11.1%), but the difference was nonsignificant (*P* = 0.172) (Table 3). The clinical pregnancy rate was higher in the euploidy group than the aneuploidy group only among the patients whose embryos had fair morphology (*P* = 0.014) (Additional file 1: Table S4). In addition, the proportion of embryos with good morphology was highest in the chaotic abnormal/NA embryo group (54.7%) compared with the other two groups (Table 4).

**Table 3:** Morphological grade-stratified comparison of clinical outcomes

Quality grade	NICS results	Ratio	Clinical pregnancy rate	Miscarriage rate	Ongoing pregnancy rate	Live birth rate
Good (n = 79)	Euploid	41.8% (33/79)	63.6% (21/33)	9.5% (2/21)	57.6% (19/33)	57.6% (19/33)
	Aneuploid	21.5% (17/79)	35.3% (6/17)	33.3% (2/6)	23.5% (4/17)	23.5% (4/17)
	Chaotic/NA	36.7% (29/79)	69.0% (20/29)	20.0% (4/20)	55.2% (16/29)	55.2% (16/29)
	p-value		0.066	0.253	0.054	0.054
Fair (n = 81)	Euploid	42.0% (34/81)	55.9% (19/34)	10.5% (2/19)	50.0% (17/34)	50.0% (17/34)
	Aneuploid	34.6% (28/81)	25.0% (7/28)	14.3% (1/7)	21.4% (6/28)	21.4% (6/28)
	Chaotic/NA	23.5% (19/81)	52.6% (10/19)	10.0% (1/10)	47.4% (9/19)	47.4% (9/19)
	p-value		0.037	0.999	0.053	0.053
Poor (n = 50)	Euploid	44.0% (22/50)	45.5% (10/22)	40.0% (4/10)	27.3% (6/22)	22.7% (5/22)
	Aneuploid	46.0% (23/50)	30.4% (7/23)	14.3% (1/7)	21.7% (5/23)	21.7% (5/23)
	Chaotic/NA	10.0% (5/50)	40.0% (2/5)	50.0% (1/2)	20.0% (1/5)	20.0% (1/5)
	p-value		0.643	0.381	0.891	0.999
P value for quality grade subgroups		0.005	0.038 (59.5% vs 44.4% vs 38.0%)	0.172 (17.0% vs 11.1% vs 31.6%)	0.016 (49.4% vs 39.5% vs 24.0%)	0.008 (49.4% vs 39.5% vs 22.0%)

**Table 4:** Distribution of morphological grades among euploid, aneuploid, and chaotic abnormal/NA embryos

NICS results	Quality grade	Numbers	Ratio
Euploid group (n = 89)	Good	33	37.1%
	Fair	34	38.2%
	Poor	22	24.7%
Aneuploid group (n = 68)	Good	17	25.0%
	Fair	28	41.2%
	Poor	23	33.8%
Chaotic/NA group (n = 53)	Good	29	54.7%
	Fair	19	35.8%
	Poor	5	9.4%

According to embryonic age, patients were divided into two groups of day 5 (D5) and D6 blastocysts for stratified analysis. Compared to the D6 blastocyst group, the D5 blastocyst group had a nonsignificantly different euploidy rate (40.4% versus 48.1%,* P* = 0.320) but a significantly higher percentage of chaotic abnormal/NA embryos (30.1% *versus* 11.1%,* P* = 0.006) (Table 5). In addition, the overall clinical pregnancy rate, ongoing pregnancy rate, and live birth rate were significantly higher in the D5 blastocyst group than in the D6 blastocyst group (57.7% versus 22.2%,* P* < 0.001; 48.1% versus 14.8%,* P* < 0.001; and 48.1% versus 13.0%,* P* < 0.001, respectively) (Table 5). In the patients with D5 blastocyst transfer, significant differences were found in the clinical pregnancy rate, ongoing pregnancy rate, and live birth rate among the euploidy group, the aneuploidy group, and the chaotic abnormal/NA embryo group (*P* = 0.008,* P* = 0.002, and* P* = 0.002, respectively) (Table 5). All three parameters in the euploidy group were significantly higher than those in the aneuploidy group (*P* = 0.002,* P* = 0.001, and* P* = 0.001) (Additional file 1: Table S5). In the patients with D6 blastocyst transfer, the live birth rate varied significantly among the euploidy group, the aneuploidy group, and the chaotic abnormal/NA embryo group (7.7% versus 9.1% versus 50.0%,* P* = 0.046) (Table 5). However, pairwise comparisons didn’t show significant differences, which may be related to the small sample size (Additional file 1: Table S6).

**Table 5:** Embryonic days -stratified comparison of clinical outcomes

Embryonic days	NICS results	Ratio	Clinical pregnancy rate	Miscarriage rate	Ongoing pregnancy rate	Live birth rate
D5 (n = 156)	Euploid	40.4%(63/156)	68.3%(43/63)	9.3%(4/43)	61.9%(39/63)	61.9%(39/63)
	Aneuploid	29.5%(46/156)	39.1%(18/46)	22.2%(4/18)	28.3%(13/46)	28.3%(13/46)
	Chaotic/NA	30.1%(47/156)	61.7%(29/47)	20.7%(6/29)	48.9%(23/47)	48.9%(23/47)
	p-value		0.008	0.242	0.002	0.002
D6 (n = 54)	Euploid	48.1%(26/54)	26.9%(7/26)	57.1%(4/7)	11.5%(3/26)	7.7%(2/26)
	Aneuploid	40.7%(22/54)	9.1%(2/22)	0(0/2)	9.1%(2/22)	9.1%(2/22)
	Chaotic/NA	11.1%(6/54)	50.0%(3/6)	0(0/3)	50.0%(3/6)	50.0%(3/6)
	p-value		0.055	0.180	0.072	0.046
P value for embryonic days subgroups		0.019	< 0.001(57.7%vs 22.2%)	0.217(15.6%vs33.3%)	< 0.001(48.1%vs14.8%)	< 0.001(48.1%vs13.0%)

### Relationship between the fertilization approaches and clinical outcomes

Clinical guidelines recommend intracytoplasmic sperm injection (ICSI) fertilization to patients who undergo PGT, while controversies remain regarding whether PGT is applicable in patients undergoing IVF. Therefore, the patients included in this study were divided into the IVF and ICSI groups according to fertilization approach. The results showed no statistically significant differences in clinical outcomes between the two groups (*P* > 0.05). In patients who received IVF, the ongoing pregnancy and live birth rates were statistically significantly different among the euploidy, aneuploidy and chaotic/NA embryo groups (*P* = 0.025, *P* = 0.025, respectively). In patients who received ICSI, the clinical pregnancy and live birth rates were also statistically significantly different among those three groups (*P* = 0.003, *P* = 0.050, respectively) (Table 6). Pairwise comparison revealed that among IVF patients, the ongoing pregnancy and live birth rates of the chaotic/NA embryo group were significantly higher than those of the aneuploidy group (*P* = 0.016 and *P* = 0.016, respectively) (Additional file 1: TableS7). Among ICSI patients, the euploidy group and the chaotic abnormal/NA embryo group had significantly higher clinical pregnancy rates than the aneuploidy group (*P* = 0.001 and *P* = 0.003, respectively) (Additional file 1: Table S8).

**Table 6:** Approaches of fertilization -stratified comparison of clinical outcomes

Approaches of fertilization	NICS results	Ratio	Clinical pregnancy rate	Miscarriage rate	Ongoing pregnancy rate	Live birth rate
IVF (n = 111)	Euploid	34.2%(38/111)	57.9%(22/38)	13.6%(3/22)	50.0%(19/38)	50.0%(19/38)
	Aneuploid	38.7%(43/111)	37.2%(16/43)	25.0%(4/16)	25.6%(11/43)	25.6%(11/43)
	Chaotic/NA	27.0%(30/111)	63.3%(19/30)	15.8%(3/19)	53.3%(16/30)	53.3%(16/30)
	p-value		0.055	0.681	0.025	0.025
ICSI (n = 99)	Euploid	51.5%(51/99)	54.9%(28/51)	17.9% (5/28)	45.1%(23/51)	43.1%(22/51)
	Aneuploid	25.3%(25/99)	16.0%(4/25)	0(0/4)	16.0%(4/25)	16.0%(4/25)
	Chaotic/NA	23.2%(23/99)	56.5%(13/23)	23.1%(3/13)	43.5%(10/23)	43.5%(10/23)
	p-value		0.003	0.721	0.038	0.050
P value for approaches of fertilization subgroups		0.031	0.393(51.4%vs 45.5%)	0.975(17.5%vs17.8%)	0.547(41.4%vs37.4%)	0.451(41.4%vs36.4%)

## Discussion

In this study, we retrospectively analysed and compared the NICS data of 210 frozen-thawed blastocyst culture medium samples and the corresponding clinical outcomes of patients who received SBT with morphologically good-quality embryos. The results showed that the euploidy group had significantly higher clinical pregnancy, ongoing pregnancy, and live birth rates than the aneuploid group (56.2% versus 29.4%, adjusted OR 0.33, 95% CI 0.15–0.72; 47.2% versus 22.1%, adjusted OR 0.34, 95% CI 0.15–0.77; 46.1% versus 22.1%, adjusted OR 0.39, 95% CI 0.18–0.86, respectively), but this group showed nonsignificant differences in the three parameters compared with the chaotic abnormal/NA embryo group (56.2% versus 60.4%, adjusted or 0.85, 95% CI 0.39–1.85; 47.2% versus 49.1%, adjusted or 0.76, 95% CI 0.36–1.63; 46.1% versus 49.1%, adjusted or 0.78, 95% CI 0.37–1.67, respectively) (Fig. 2 and Additional file 1: Table S2), suggesting that the patients who were implanted with euploid embryos had more satisfactory clinical outcomes than those implanted with aneuploid embryos, which is consistent with findings from previous studies [31, 32]. However, the aneuploidy group had a live birth rate of 22.1%, indicating that either the test results were false positive or the embryos had the ability to repair themselves. In addition, the chaotic abnormal/NA embryo group was not significantly different from the euploidy group in clinical pregnancy, ongoing pregnancy, and live birth rates (56.2% versus 60.4%, adjusted or 0.85, 95% CI 0.39–1.85; 47.2% versus 49.1%, adjusted or 0.76, 95% CI 0.36–1.63; 46.1% versus 49.1%, adjusted or 0.78, 95% CI 0.37–1.67, respectively), possibly because this group had a high proportion of morphologically ‘good’ embryos (good: 54.7%; fair:37.1%; poor:25.0%) (Table 4). A ‘good’ embryo has a dense cell arrangement and might release less DNA into culture medium, resulting in test failure or indeterminate results. Magli et al. [33] also found that transferring an embryo with successful blastocoel fluid amplification led to a clinical pregnancy rate of only 37% and an ongoing pregnancy rate of 18%, while transferring an embryo with blastocoel fluid amplification failure resulted in a clinical pregnancy rate of 77% and an ongoing pregnancy rate of 70%. Therefore, in cases where euploid embryos are unavailable, a possible solution is to consider embryos with high morphological quality but test failure by sequencing if the patient provides informed consent after fully understanding the risk.

In this study, frozen embryos were thawed and cultured following a routine protocol in our centre, i.e., thawed blastocysts were cultured in microdrops (15–20 µL) for 6 h, and SCMs were collected for embryo chromosomal genetic testing. In a previous study conducted by Kuznyetsov et al. [17], a mixture of blastocoel fluid and culture medium collected after frozen embryos were thawed and cultured in a 25 µL culture system for 24 h had an amplification success rate of 100%, and the NICS results had a concordance rate of 96.4% with whole-embryo tests. In the study of Huang et al. [18], in which frozen embryos were thawed and cultured in a 15 µL culture system for 24 h, the SCMs had an amplification efficiency of 92.3%, and the NICS results had a concordance rate of 93.8% with whole-embryo tests. Jiao et al. [19] reported that after frozen embryos were thawed and cultured in a 12-µL culture system for 15 h, a mixture of blastocoel fluid and culture medium had an amplification efficiency of 100%, and the NICS results had a concordance rate of 90.48% with whole-embryo tests. In a recent study by Li et al. [20], after 41 frozen embryos classified as mosaics were thawed and cultured in a 15 µL culture system for 14–18 h, the culture medium, TE cells, and remaining whole embryos were collected for NICS and PGT-A. The results showed that 85.4% of the whole embryos were euploidy, 82.9% of which were reported to be euploidy by NICS [20]. The studies above showed that niPGT-A has the potential for embryo chromosomal screening. However, in clinical practice, the total thawing and culture duration of frozen embryos is generally 2–3 h [21, 34] such that an embryo that has developed into a blastocyst can be implanted within the optimal time window to improve the success rate of implantation. In the present study, we thawed and cultured frozen embryos for 6 h following a routine protocol in our centre, which can fully activate the developmental potential of frozen-thawed embryos, facilitate embryo implantation, and meet the sample size requirement for NICS.

Before the sequencing depth of the samples was confirmed, the raw reads of 53 samples at different depths were analyzed. The sequencing reads were reduced to: 200 K, 300 K, 400 K, 500 K, 600 K, 800 K, 1 M. Among the 53 samples with ≥ 10 Mb duplications or deletions, when the reads reached 400 k or more, the CNV results obtained by the analysis are consistent for the same sample. The consistency rate is 100% (53/53). Among the 29 samples with ≥ 10 Mb duplications or deletions and 30–70% mosaicism, when the reads reached 400 K or more, the CNV results obtained by the analysis are consistent for the same sample. The consistency rate is also 100% (29/29) (Additional file 2: Figure S1). These results showed that the CNV accuracy can be credible when the amount of sequencing data for each sample must reach 400 K. In addition, we also refer to the sequencing depth of the recently published articles on NICS [19, 20, 25–27]. Finally, the sequencing reads in this study were confirmed to be ~ 2 M.

To further explore the factors affecting clinical outcomes, we performed stratified analyses for female age, morphological grade, and embryonic days. The results showed that the embryo ploidy rate decreased with increasing female age (45.2% in the < 35 age group and 31.0% in the ≥ 35 age group), and the clinical pregnancy, ongoing pregnancy, and live birth rates also showed similar declines (55.4% versus 21.4%, 45.8% versus 14.3%, 45.2% versus 14.3%, respectively) (Table 2), which is consistent with the results from previous studies [31, 32]. Compared with D6 blastocysts, D5 blastocysts did not show any significant difference in the euploidy rate (40.4% versus 48.1%, *p* = 0.320) but resulted in significantly increased clinical pregnancy, ongoing pregnancy, and live birth rates (57.7% versus 22.2%; 48.1% versus 14.8%; 48.1% versus 13.0%, respectively) (Table 5). Kovalevsky et al. [35] reported that patients with D5 frozen embryo transfer had significantly higher clinical pregnancy and ongoing pregnancy rates than those implanted with D6 frozen embryos. In the present study, D5 embryos had a slightly lower euploidy rate (40.4% versus 48.1%) but a higher chaotic/NA rate than D6 embryos (30.1% versus 11.1%), possibly because in D5 embryos, good embryos were significantly higher than D6 (p < 0.001, Additional file 1: Table S9). Good embryos have dense cells and release less DNA into culture medium during thawing and warming, leading to a higher chaotic abnomal/NA rate.

Based on the NICS results, the percentage of aneuploid embryos was higher among the embryos with a worse morphology, as evidenced by the data showing that aneuploid embryos accounted for 21.5% of morphologically good-quality embryos, 34.6% of fair-quality embryos, and 46.0% of poor-quality embryos(*P* = 0.013).The overall clinical pregnancy, ongoing pregnancy, and live birth rates of patients decreased with morphological quality deterioration of embryos (59.5% versus 44.4% versus 38.0%,* P* = 0.038; 49.4%versus 39.5% versus 24.0%,* P* = 0.016; 49.4%versus 39.5% versus 22.0%,* P* = 0.008) (Table 3). Peng et al. [36] found that the morphological development of embryos had a positive effect on the pregnancy and live birth rates but not on miscarriage rates in patients with euploid embryo transfer. In the present study, the live birth rate was highest (57.6%) in patients implanted with morphologically ‘good’ euploid embryos (Table 3). Therefore, euploid embryos with a ‘good’ morphology should be the first choice for implantation. According to the live birth rate, we recommend that the ideal embryo for transfer is ‘euploid embryo with good morphology’, followed sequentially by ‘chaotic abnormal/NA embryos with good morphology’, ‘euploid embryo with fair morphology’, and ‘chaotic abnormal/NA embryos with fair morphology’ (Table 3).

In addition, this study included IVF-fertilized embryos. Previous research has shown that the lysis conditions for WGA of biopsied cells (polar bodies, blastomeres, or TE cells) might be too mild to amplify sperm DNA, whereas the amplification of single-sperm DNA requires strong lysis conditions, and therefore, PGT might be suitable for IVF-fertilized embryos [37]. In the present study, no significant difference in clinical outcomes was found between patients who received ICSI and IVF. De Munck et al. [38] also reported that PGT-A did not result in any differences in the blastocyst formation rate, total number of blastocysts, and euploidy rate between embryos fertilized by ICSI and IVF. Therefore, niPGT-A may be applicable regardless of fertilization approaches such that both fertilization procedures and chromosomal screening will be noninvasive in the future.

This study also has some limitations. To avoid the impact of refreezing on the embryos, the embryos must be implanted during the current frozen-thawed cycle. In our protocol, the estimated time for thawing, testing, and implanting is at least 15 h. Therefore, our method is most applicable for early blastocysts frozen in stage 3 or 4, which allows sufficient time for testing, and then the blastocysts can be transferred directly without being frozen again. However, the method is not recommended for blastocysts frozen at stages 5–6. The long culture time might cause the embryos that have developed into blastocysts to miss the optimal time window for implantation and embryo hatching, leading to a lower success rate of implantation.

In conclusion, this study is the first large-scale retrospective clinical study to analyse the relationship between NICS results and clinical outcomes in patients implanted with single frozen-thawed blastocysts selected based on morphological quality. The results showed that in clinical practice, frozen embryos could be thawed and cultured in 15–20 µL of culture medium for 6 h, and the culture medium was collected for NICS prior to embryo transfer. The clinical outcomes of patients implanted with euploid embryos were significantly better than those of patients implanted with aneuploidy embryos but did not differ from those of patients implanted with chaotic abnormal/NA embryos. NICS combined with morphological grading can be clinically used to select blastocysts for transfer in frozen-thawed cycles. Embryo suitability for transfer is in the order of ‘euploid embryo with good morphology’, ‘chaotic abnormal/NA embryo with good morphology’, ‘euploid embryo with fair morphology’, and ‘chaotic abnormal/NA embryo with fair morphology’(Table 3 and Additional file 2: Figure S2). Meanwhile, the clinical outcomes of patients were not related to the fertilization approach in the niPGT-A cycle, which might provide patients with a new treatment strategy where both the fertilization approach and PGT can be noninvasive.

## Supplementary Information

**Additional file 1: Table S1.** The comparison of CNV results of abortions and NICS results of embryos. **Table S2.** Logistic regression analysis compared the clinical outcomes of the euploidy group, the aneuploidy group, and the chaotic abnormal/NA embryos group. **TableS3.** Pairwise comparison of clinical pregnancy, ongoing pregnancy rates and live birth rate among female patients younger than 35 years. **Table S4.** Pairwise comparison of clinical pregnancy rates among patients implanted with morphologically ‘fair’ embryos. **Table S5.** Pairwise comparison of clinical pregnancy, ongoing pregnancy, and live birth rates among patients implanted with D5 embryos. **Table S6.** Pairwise comparison of live birth rates among patients implanted with D6 embryos. **Table S7.** Pairwise comparison of ongoing pregnancy rate and live birth rate among patients received conventional IVF. **Table S8.** Pairwise comparison of clinical pregnancy rate, ongoing pregnancy rate and live birth rate among patients received ICSI. **Table S9.** Baseline clinical characteristics of the patients undergoing D5 and D6 embryo transfer.

**Additional file 2**: **Figure S1.** The consistency rate in different sequencing reads. **Figure S2.** Priority embryo transfer sequence.

## Data Availability

The datasets used and/or analysed during the current study are available from the corresponding author on reasonable request.

## Abbrevations

SCM: Spent culture media
NICS: Noninvasive chromosomal screening
eSBT: Elective single-blastocyst transfer
IVF-ET: In vitro fertilization-embryo transfer
PGT-A: Preimplantation genetic testing for aneuploidy
TE: Trophectoderm
CNV: Copy number variation
WGA: Whole-genome amplification
NGS: Next-generation sequencing
BMI: Body mass index
ICSI: Intracytoplasmic sperm injection
