# Supplementary material for: Clinical application of noninvasive chromosomal screening for elective single-blastocyst transfer in frozen-thawed cycles
Source: J Transl Med. 2022 Dec 3;20:553. doi: 10.1186/s12967-022-03640-z (PMC9719190; doi:10.1186/s12967-022-03640-z)
Supplement: Supplementary file 1 — Additional file 1: Table S1. The comparison of CNV results of abortions and NICS results of embryos. Table S2. Logistic regression analysis compared the clinical outcomes of the euploidy group, the aneuploidy group, and the chaotic abnormal/NA embryos group. TableS3. Pairwise comparison of clinical pregnancy, ongoing pregnancy rates and live birth rate among female patients younger than 35 years. Table S4. Pairwise comparison of clinical pregnancy rates among patients implanted with morphologically ‘fair’ embryos. Table S5. Pairwise comparison of clinical pregnancy, ongoing pregnancy, and live birth rates among patients implanted with D5 embryos. Table S6. Pairwise comparison of live birth rates among patients implanted with D6 embryos. Table S7. Pairwise comparison of ongoing pregnancy rate and live birth rate among patients received conventional IVF. Table S8. Pairwise comparison of clinical pregnancy rate, ongoing pregnancy rate and live birth rate among patients received ICSI. Table S9. Baseline clinical characteristics of the patients undergoing D5 and D6 embryo transfer. [file 12967_2022_3640_MOESM1_ESM.docx]

**Supplementary Tables**

**Additional file 1: Table 1.** The comparison of CNV results of abortions and NICS results of embryos.

| Number | NICS | Abortions | Group |
| --- | --- | --- | --- |
| 1 | 46,XN,-4p(×1,mos),+5p(×3,mos),+8(×3,mos),-10p(×1,mos),-14(×1,mos),-16(×1,mos),+20(×3,mos),+21(×3,mos) | 46,XN | Chaotic abnomal/NA; Euploid |
| 2 | 46,XN | 46,XN | Euploid; Euploid |
| 3 | 46,XN | 46,XN | Euploid; Euploid |
| 4 | 46,XN,+7p(×3,mos),+10q(×3,mos),+16(×3),+17p(×3,mos),+17q(×3,mos),-18p(×1,mos),-19p(×1,mos) | 46,XN | Chaotic abnomal/NA; Euploid |
| 5 | 46,XN,+16p(×3,mos),+16q(×3,mos) | 47,XN,+16(×3) | Aneuploid; Aneuploid |
| 6 | 46,XY,+Xp(×2,mos),+Xq(×2,mos),-8q(×1,mos),+13(×3,mos),-19p(×1,mos),-21(×1,mos) | 46,XN | Aneuploid; Euploid |

**Additional file 1: Table 2.** Logistic regression analysis compared the clinical outcomes of the euploidy group, the aneuploidy group, and the chaotic abnormal/NA embryos group.

|  | **Euploidy group** | **Aneuploidy group** | **Chaotic abnormal/NA embryos group** | **Aneuploid group vs Euploid group** | **Chaotic abnormal/NA embryos group vs Euploid group** |
| --- | --- | --- | --- | --- | --- |
|  |  |  |  | **Adjusted odds ratio (95% CI), p-value ^a^** | **Adjusted odds ratio (95% CI), p-value ^a^** |
| Clinical pregnancy rate | 56.2% (50/89) | 29.4% (20/68) | 60.4% (32/53) | 0.33(0.15-0.72),0.005 | 0.85(0.39-1.85),0.689 |
| Miscarriage rate | 16.0% (8/50) | 20.0% (4/20) | 18.8% (6/32) | 0.58(0.16-2.08),0.401 | 1.33(0.41-4.24),0.635 |
| Ongoing pregnancy rate | 47.2% (42/89) | 22.1% (15/68) | 49.1% (26/53) | 0.34(0.15-0.77),0.009 | 0.76(0.36-1.63),0.486 |
| Live birth rate | 46.1% (41/89) | 22.1% (15/68) | 49.1% (26/53) | 0.39(0.18-0.86),0.019 | 0.78(0.37-1.67),0.520 |

^a^ Logistic regression analysis adjusted for male and female age, morphological grade, embryonic days, fertilization approaches, and infertility type.

**Additional file 1: Table3.** Pairwise comparison of clinical pregnancy, ongoing pregnancy rates and live birth rate among female patients younger than 35 years.

| **Age of female** | **Groups** | **Clinical pregnancy rate** | **P-value** | **Ongoing pregnancy rate** | **P-value** | **Live birth rate** | **P-value** |
| --- | --- | --- | --- | --- | --- | --- | --- |
| <35 year  (n=168) | Euploid vs Aneuploid | 60.5% vs 38.6% | 0.021 | 51.3% vs 29.5% | 0.020 | 50.0% vs 29.5% | 0.029 |
|  | Chaotic/NA vs Aneuploid | 62.5% vs 38.6% | 0.022 | 52.1% vs 29.5% | 0.028 | 52.1% vs 29.5% | 0.028 |
|  | Euploid vs Chaotic/NA | 60.5% vs 62.5% | 0.826 | 51.3% vs 52.1% | 0.934 | 50.0% vs 52.1% | 0.821 |

P-value equal to or less than 0.017 was considered to be statistically significant.

**Additional file 1: Table 4.** Pairwise comparison of clinical pregnancy rates among patients implanted with morphologically ‘fair’ embryos.

| **Quality grade** | **Groups** | **Clinical pregnancy rate** | **P-value** |
| --- | --- | --- | --- |
| Fair  (n=81) | Euploid vs Aneuploid | 55.9% vs 25.0% | 0.014 |
|  | Chaotic/NA vs Aneuploid | 52.6% vs 25.0% | 0.053 |
|  | Euploid vs Chaotic/NA | 55.9% vs 52.6% | 0.820 |

P-value equal to or less than 0.017 was considered to be statistically significant.

**Additional file 1: Table 5.** Pairwise comparison of clinical pregnancy, ongoing pregnancy, and live birth rates among patients implanted with D5 embryos.

| **Embryo stage** | **Groups** | **Clinical pregnancy rate** | **P-value** | **Ongoing pregnancy rate** | **P-value** | **Live birth rate** | **P-value** |
| --- | --- | --- | --- | --- | --- | --- | --- |
| D5  (n=156) | Euploid vs Aneuploid | 68.3% vs 39.1% | 0.002 | 61.9% vs 28.3% | 0.001 | 61.9% vs 28.3% | 0.001 |
|  | Chaotic/NA vs Aneuploid | 61.7% vs 39.1% | 0.030 | 48.9% vs 28.3% | 0.041 | 48.9% vs 28.3% | 0.041 |
|  | Euploid vs Chaotic/NA | 68.3% vs 61.7% | 0.475 | 61.9% vs 48.9% | 0.175 | 61.9% vs 48.9% | 0.175 |

P-value equal to or less than 0.017 was considered to be statistically significant.

**Additional file 1: Table 6.** Pairwise comparison of live birth rates among patients implanted with D6 embryos.

| **Embryo stage** | **Groups** | **Live birth rate** | **P-value** |
| --- | --- | --- | --- |
| D6  (n=54) | Euploid vs Aneuploid | 7.7% vs 9.1% | 0.999 |
|  | Chaotic/NA vs Aneuploid | 50.0% vs 9.1% | 0.050 |
|  | Euploid vs Chaotic/NA | 7.7% vs 50.0% | 0.034 |

P-value equal to or less than 0.017 was considered to be statistically significant.

**Additional file 1: Table 7.** Pairwise comparison of ongoing pregnancy rate and live birth rate among patients received conventional IVF.

| **Fertilization method** | **Groups** | **Ongoing pregnancy rate** | **P-value** | **Live birth rate** | **P-value** |
| --- | --- | --- | --- | --- | --- |
| IVF  (n=111) | Euploid vs Aneuploid | 50.0% vs 25.6% | 0.023 | 50.0% vs 25.6% | 0.023 |
|  | Chaotic/NA vs Aneuploid | 53.3% vs 25.6% | 0.016 | 53.3% vs 25.6% | 0.016 |
|  | Euploid vs Chaotic/NA | 50.0% vs 53.3% | 0.785 | 50.0% vs 53.3% | 0.785 |

P-value equal to or less than 0.017 was considered to be statistically significant.

**Additional file 1: Table 8.** Pairwise comparison of clinical pregnancy rate, ongoing pregnancy rate and live birth rate among patients received ICSI.

| **Fertilization method** | **Groups** | **Clinical pregnancy rate** | **P-value** | **Ongoing pregnancy rate** | **P-value** | **Live birth rate** | **P-value** |
| --- | --- | --- | --- | --- | --- | --- | --- |
| ICSI  (n=99) | Euploid vs Aneuploid | 54.9% vs 16.0% | 0.001 | 45.1% vs 16.0% | 0.013 | 43.1% vs 16.0% | 0.019 |
|  | Chaotic/NA vs Aneuploid | 56.5% vs 16.0% | 0.003 | 43.5% vs 16.0% | 0.036 | 43.5% vs 16.0% | 0.036 |
|  | Euploid vs Chaotic/NA | 54.9% vs 56.5% | 0.897 | 45.1% vs 43.5% | 0.897 | 43.1% vs 43.5% | 0.978 |

P-value equal to or less than 0.017 was considered to be statistically significant.

**Additional file 1: Table 9. Baseline clinical characteristics of the patients undergoing D5 and D6 embryo transfer.**

| **Characteristic** | **D5** | **D6** | **P-value** |
| --- | --- | --- | --- |
| Number of patients, n | 156 | 54 |  |
| Age of female, mean±SD, y | 31.2±4.3 | 32.17±3.8 | 0.164 |
| BMI of female, mean±SD, kg/m^2^ | 22.1±3.5 | 22.0±3.7 | 0.850 |
| Age of male, mean±SD, y | 33.8±5.1 | 34.3±4.1 | 0.497 |
| BMI of male, mean±SD, kg/m^2^ | 24.7±3.5 | 23.9±3.5 | 0.142 |
| Infertility duration, mean±SD, y | 4.0±2.9 | 4.4±3.1 | 0.383 |
| Types of infertility | | |  |
| Primary (%) | 58.3%(91/156) | 51.9%(28/54) | 0.407 |
| Secondary (%) | 41.7%(65/156) | 48.1%(26/54) |  |
| Indication | |  |  |
| Male factor (%) | 30.8%(48/156) | 31.5%(17/54) | 0.995 |
| Female factor (%) | 37.2%(58/156) | 37.0%(20/54) |  |
| Both (%) | 32.1%(50/156) | 31.5%(17/54) |  |
| Approaches of fertilization | | |  |
| ICSI (%) | 44.2%(69/156) | 55.6%(30/54) | 0.151 |
| IVF(%) | 55.8%(87/156) | 44.4%(24/54) |  |
| Quality Grade | | |  |
| Good (AA/BA/AB) (%) | 47.4%(74/156) | 9.3%(5/54) | <0.001 |
| Fair (BB/AC) (%) | 39.7%(62/156) | 35.2%(19/54) |  |
| Poor (CA/BC/CB) (%) | 12.8%(20/156) | 55.6%(30/54) |  |
