# Supplementary material for: Clinical application of noninvasive chromosomal screening for elective single-blastocyst transfer in frozen-thawed cycles
Source: J Transl Med. 2022 Dec 3;20:553. doi: 10.1186/s12967-022-03640-z (PMC9719190; doi:10.1186/s12967-022-03640-z)
Supplement: Supplementary file 2 — Additional file 2: Figure S1. The consistency rate in different sequencing reads. Figure S2. Priority embryo transfer sequence. [file 12967_2022_3640_MOESM2_ESM.docx]

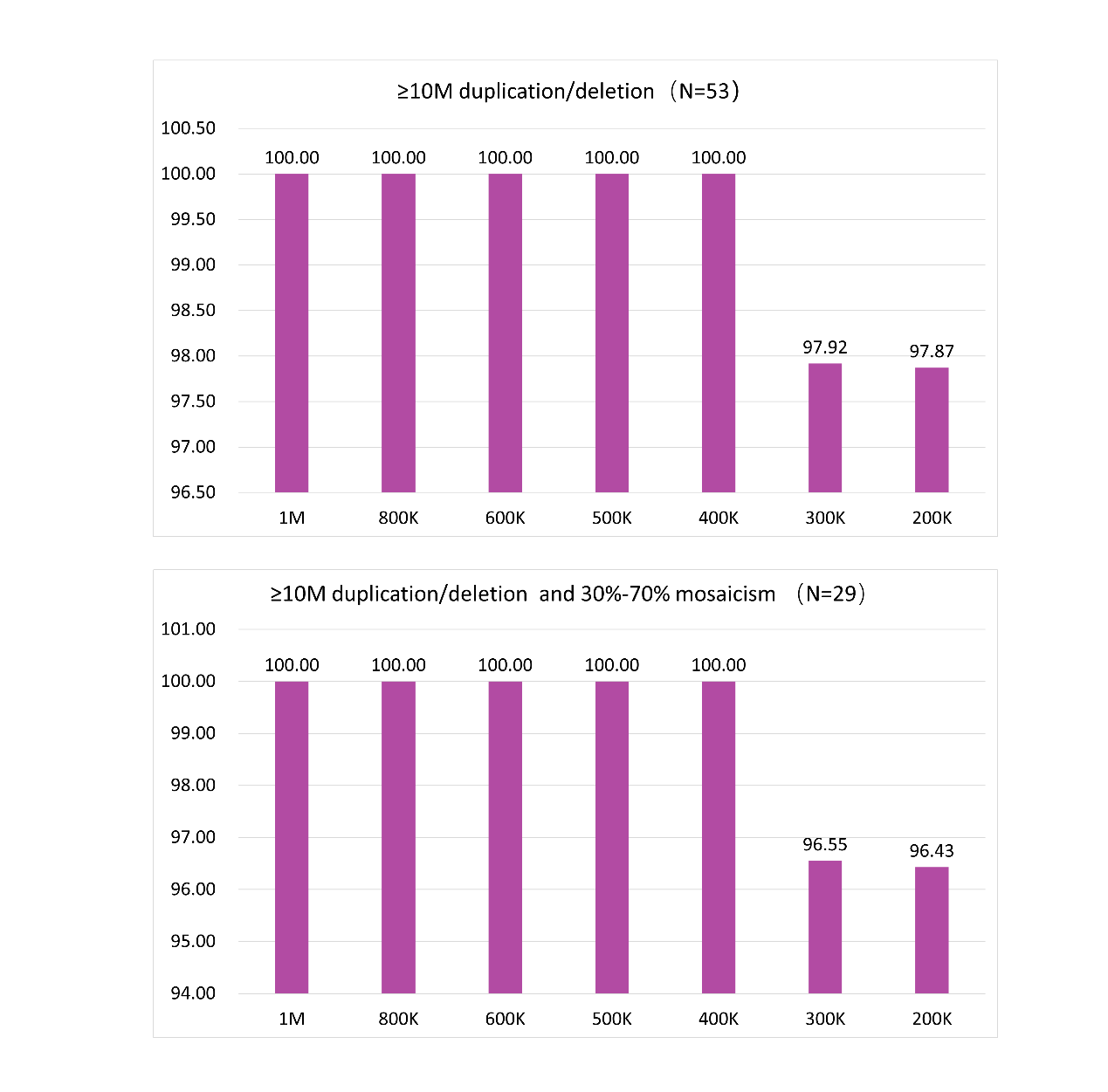


**Additional file 3: Figure S1. The consistency rate in different sequencing reads.**


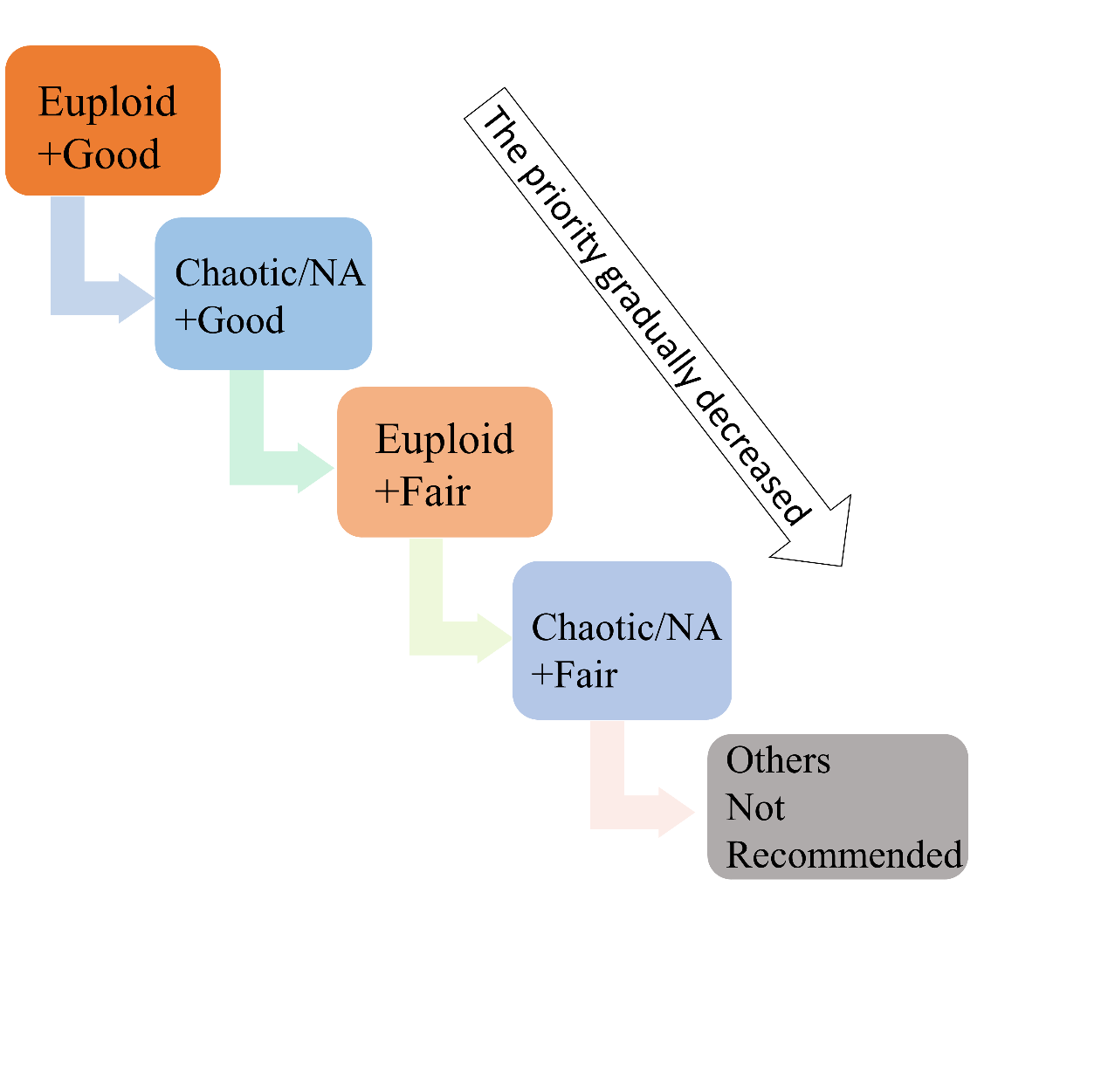


**Additional file 3: Figure S2.** Priority embryo transfer sequence.
